# Intravenous Sodium Thiosulphate for Calciphylaxis of Chronic Kidney Disease: A Systematic Review and Meta-analysis

**DOI:** 10.1001/jamanetworkopen.2023.10068

**Published:** 2023-04-26

**Authors:** Wen Wen, Ignacio Portales-Castillo, Rituvanthikaa Seethapathy, Olivia Durant, Beza Mengesha, Scott Krinsky, Daniela Kroshinsky, Sahir Kalim, Jeremy Goverman, Rosalynn M. Nazarian, Vipul Chitalia, Rajeev Malhotra, Rafael Kramann, Cindy K. Malhotra, Sagar U. Nigwekar

**Affiliations:** 1 Department of Nephrology, Beijing Tsinghua Changgung Hospital, School of Clinical Medicine, Tsinghua University, Beijing, China; 2 Division of Nephrology, Massachusetts General Hospital, Boston, Massachusetts; 3 Bouvé College of Health Sciences, Northeastern University, Boston, Massachusetts; 4 Department of Dermatology, Massachusetts General Hospital, Boston; 5 Sumner Redstone Burn Center, Massachusetts General Hospital, Boston; 6 Department of Pathology, Massachusetts General Hospital, Boston; 7 Renal Section, Department of Medicine, Boston University Medical Center, Boston, Massachusetts; 8 Cardiovascular Research Center and the Cardiology Division of the Department of Medicine, Massachusetts General Hospital and Harvard Medical School, Boston; 9 Division of Nephrology and Clinical Immunology, Medical Faculty RWTH Aachen University, Aachen, Germany; 10 Institute of Experimental Medicine and Systems Biology, Medical Faculty RWTH Aachen University, Aachen, Germany; 11 Department of Internal Medicine, Nephrology and Transplantation, Erasmus Medical Center, Rotterdam, the Netherlands; 12 Department of Pharmacy, Massachusetts General Hospital, Boston

## Abstract

**Importance:**

Calciphylaxis is a rare disease with high mortality mainly involving patients with chronic kidney disease (CKD). Sodium thiosulphate (STS) has been used as an off-label therapeutic in calciphylaxis, but there is a lack of clinical trials and studies that demonstrate its effect compared with those without STS treatment.

**Objective:**

To perform a meta-analysis of the cohort studies that provided data comparing outcomes among patients with calciphylaxis treated with and without intravenous STS.

**Data Sources:**

PubMed, Embase, Cochrane Library, Web of Science, and ClinicalTrials.gov were searched using relevant terms and synonyms including *sodium thiosulphate* and calci* without language restriction.

**Study Selection:**

The initial search was for cohort studies published before August 31, 2021, that included adult patients diagnosed with CKD experiencing calciphylaxis and could provide a comparison between patients treated with and without intravenous STS. Studies were excluded if they reported outcomes only from nonintravenous administration of STS or if the outcomes for CKD patients were not provided.

**Data Extraction and Synthesis:**

Random-effects models were performed. The Egger test was used to measure publication bias. Heterogeneity was assessed using the *I*^2^ test.

**Main Outcomes and Measures:**

Skin lesion improvement and survival, synthesized as ratio data by a random-effects empirical Bayes model.

**Results:**

Among the 5601 publications retrieved from the targeted databases, 19 retrospective cohort studies including 422 patients (mean age, 57 years; 37.3% male) met the eligibility criteria. No difference was observed in skin lesion improvement (12 studies with 110 patients; risk ratio, 1.23; 95% CI, 0.85-1.78) between the STS and the comparator groups. No difference was noted for the risk of death (15 studies with 158 patients; risk ratio, 0.88; 95% CI, 0.70-1.10) and overall survival using time-to-event data (3 studies with 269 participants; hazard ratio, 0.82; 95% CI, 0.57-1.18). In meta-regression, lesion improvement associated with STS negatively correlated with publication year, implying that recent studies are more likely to report a null association compared with past studies (coefficient = −0.14; *P* = .008).

**Conclusions and Relevance:**

Intravenous STS was not associated with skin lesion improvement or survival benefit in patients with CKD experiencing calciphylaxis. Future investigations are warranted to examine the efficacy and safety of therapies for patients with calciphylaxis.

## Key Points

**Question:**

Is intravenous sodium thiosulphate (STS) treatment associated with improvement in skin lesions and survival in patients with chronic kidney disease (CKD) experiencing calciphylaxis?

**Findings:**

This meta-analysis of 19 cohort studies examining STS treatment for patients with CKD experiencing calciphylaxis did not show an association between intravenous STS and skin lesion improvement or survival benefits compared with non-STS group.

**Meaning:**

These results suggest that studies of intravenous STS on patients with CKD experiencing calciphylaxis have not found an improvement in skin lesion outcomes or survival. Future studies that rigorously evaluate therapies applied to patients with calciphylaxis are urgently needed.

## Teaser

This meta-analysis of cohort studies reviews current results for the association of sodium thiosulphate treatment with skin lesions and overall survival of patients with chronic kidney disease who contract calciphylaxis.

## Introduction

Calciphylaxis is a rare but serious disorder of vascular calcification typically presenting with painful skin ulcerations that predominantly affects patients with advanced chronic kidney disease (CKD).^1^ There is no approved therapy for calciphylaxis. A 2022 meta-analysis of 6 clinical trials^2^ concluded that intravenous sodium thiosulfate (STS) could attenuate the progression of macrovascular calcification (in the coronary and iliac arteries) among hemodialysis patients. However, although STS has been used as an off-label therapeutic in calciphylaxis to improve pain and accelerate wound healing since 2004,^3^ there has been no data available from a clinical trial to inform its efficacy and safety. Many observational studies reporting the effectiveness of STS in patients with calciphylaxis lack a comparator arm, thus limiting the interpretation of their findings. To overcome this limitation, we performed a meta-analysis limited to cohort studies that provided data comparing outcomes among patients experiencing calciphylaxis treated with (intervention) and without (comparator) intravenous STS.

## Methods

### Data Sources and Search Strategy

PubMed, Embase, Cochrane Library, Web of Science, and ClinicalTrials.gov were searched using relevant terms and synonyms including *sodium thiosulphate* and calci* without language restriction. The controlled vocabulary terms, synonyms, and the complete search strategy are listed in eTables 1 and 2 in Supplement 1. We contacted the authors of eligible articles to retrieve missing data. Our search included studies published before August 31, 2021. This study was exempt from review by the Mass General Brigham institutional review board, and informed consent was not required because data were publicly available. Meta-analysis of Observational Studies in Epidemiology (MOOSE) reporting guidelines were followed. The protocol was registered and published on PROSPERO (CRD42021235860).

### Eligibility Criteria

**Figure 1.**
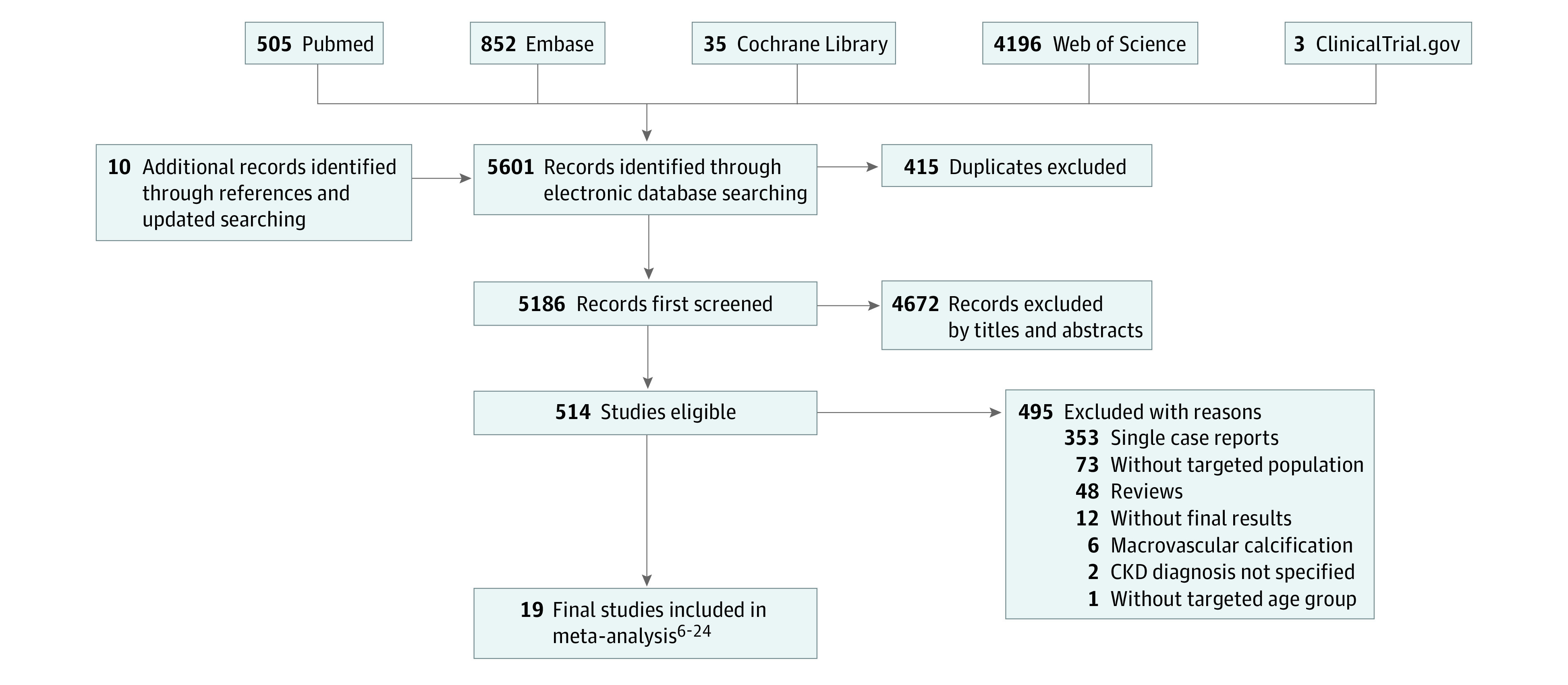
Flowchart of Study Inclusion

We searched for cohort studies that met the following criteria: (1) included adult patients (ages 18 years and older) diagnosed with CKD (defined as either kidney damage or a decreased glomerular filtration rate [GFR] of less than 60 mL/min/1.73 m^2^ for at least 3 months),^4^ (2) having calciphylaxis as the main complication studied and the primary indication for STS treatment, and (3) including both the patients treated with and without intravenous STS to provide a comparison between intervention and comparator groups (Figure 1). Studies were excluded if they reported outcomes only from nonintravenous administration of STS (eg, oral, intraperitoneal, intralesional, etc.), or the outcomes for CKD patients were not provided.

### Study Selection, Data Collection, and Risk-of-Bias Assessment

Two authors (W.W. and I.P.) independently screened the records using Endnote X9 (Clarivate) to identify eligible studies, extracted data regarding participants, intervention, and outcome measures (lesion improvement, all-cause death, and time-to-event survival), and graded the risk of bias in the studies using Risk of Bias in Non-randomized Studies of Interventions (ROBINS-I).^5^ Discrepancies among the reviewers were reevaluated by a third author (S.N.) and discussed to obtain a consensus.

### Data Synthesis

Skin lesion improvement and survival were tabulated and synthesized quantitatively by performing a random-effects empirical Bayes model. Continuous correction was automatically applied when both arms had zero events. Categorical data were analyzed using log risk ratio (RR) or log hazard ratio. Meta-regression was performed to examine the impact from publication year on treatment-related effect sizes. Funnel plot and the Egger test were used to measure the publication bias. Heterogeneity was assessed using the *I*^2^ test. An *I*^2^ index greater than 50% indicated obvious to high heterogeneity. Sensitivity analyses were performed to test effect size measurements and single studies. Stata IC version 16 (StataCorp) was used for statistical analyses. The latest update of analysis was on February 6, 2023.

## Results

Among the 5601 publications retrieved from the targeted databases, 19 retrospective cohort studies^6,7,8,9,10,11,12,13,14,15,16,17,18,19,20,21,22,23,24^ (422 patients; mean age, 57 years; 37.3% male) met our eligibility criteria. Among the 422 patients with CKD experiencing calciphylaxis, 347 were dialysis-dependent. Detailed population distribution, demographic information, and other characteristics of these cohort studies involving patients with calciphylaxis are presented in Table 1.

**Table 1.** Summary of 19 Retrospective Cohort Studies for Calciphylaxis

Source	Country	Total, No.	Kidney function cases, No.	Age, mean (SD), y	Sex, F:M	STS, No.	Longest follow-up duration	STS treatment	HBOT	Outcomes available
Slough et al,^6^ 2006	US	2	HD, 1; PD, 1	40 (1.4)	2:0	1	3 y	NA	None	Lesion improvement; mortality
Malabu et al,^7^ 2012	Australia	6	HD	66 (14.2)	4:2	3	NA	NA	2 in STS group and 2 in comparator group	Lesion improvement; mortality
Cohen et al,^8^ 2013	US	2	ESRD (1 on PD)	46 (15.6)	2:0	1	14 wk	25g with each HD session	None	Lesion improvement; mortality
Savoia et al,^9^ 2013	Italy	4	HD	71 (11.7)	2:2	3	4 y	5 g thrice weekly at the end of the dialysis	3, all in STS group	Lesion improvement; mortality
Veitch et al,^10^ 2014	UK	15	CKD stage 3, 2; HD, 11; PD, 2	47.3 (30.2)	11:4	3	9 y and 4 mo	NA	2 of the 3 patients in the STS group	Mortality
An et al,^11^ 2015	Australia	34	Kidney transplantation, 1; CKD without dialysis, 1; HD, 25; PD, 7	59.7 (11.4)	19:15	6	13 y	NA	Both groups had adequate course of HBOT	Lesion improvement
Lee et al,^12^ 2015	Singapore	12	Kidney transplantation, 1; HD, 7; PD, 4	55.8 (33.3)	10:2	11	12 mo	25 g thrice weekly	3 in STS group	Lesion improvement; mortality
McCulloch et al,^13^ 2015	US	8	ESRD on RRT	54.8 (7.4)	6:2	7	NA	NA	6 in STS group and 1 in comparator group	Lesion improvement; mortality
Loidi Pascual et al,^14^ 2016	Spain	6	CKD (1 on HD)	79.3 (7.7)	2:4	2	6 mo	NA	NA	Lesion improvement; mortality
McCarthy et al,^15^ 2016	US	63	CKD stage 5 (60 on dialysis)	NA	NA	26	NA	12.5-25 g intravenously thrice weekly for 3-6 mos	17 of the 63 cases	Mortality
Zhang et al,^16^ 2016	US	7	PD	48 (13.3)	5:2	4	1 y	25 g thrice weekly; median duration, 3.0 mo (IQR, 2.8-5.1).	57% of the 7 cases	Mortality
Ghosh et al,^17^ 2017	US	4	Kidney transplantation, 1; dialysis, 3	40.7 (16.3)	3:1	1	2 y	NA	None	Lesion improvement; mortality
Santos et al,^18^ 2017	US	117	CKD patients with secondary hyperparathyroidism	58.5 (12.8)^a^	52:42^a^	64	>12 mo	Final dose: <12.5 g, 1.7% (1); 12.5-24 g, 25.9% (15); 25-49 g, 69.0% (40); >50 g, 3.4% (2); duration: <3 mos 79.7% (47); 3-6 mso 11.9% (7); >6 mos 8.5% (5)	9 of 117 patients	Mortality
Dado et al,^19^ 2019	US	9	ESRD on RRT	50.6 (12.5)	5:4	7	265 d	25 g thrice weekly	NA	Mortality
Franco-Muñoz et al,^20^ 2019	Spain	12	CKD	NA	NA	8	NA	NA	NA	Mortality
Gaisne et al,^21^ 2020	France	89	CKD stage 4 or CKD stage 5	70 (11.1)	57:32	58	5 y	median (IQR) STS cumulative dose, 488 (300-750) g; median STS duration, 6 (4-10) wk	NA	Mortality
Saito et al,^22^ 2020	Japan	5	HD, 4; PD, 1	56.2 (10.2)	3:2	4	Average follow-up, 7.4 mo	NA	1 in STS group	Lesion improvement; mortality
Omer et al,^23^ 2021	US	24	ESRD on RRT	56.3 (14.6)	18:6	22	NA	NA	2 of all	Lesion improvement
Jiun et al,^24^ 2021	Malaysia	3	HD	40.3 (14.6)	2:1	2	6 mo	NA	None	Lesion improvement; mortality

Abbreviations: CKD, chronic kidney disease; ESRD, end-stage renal disease; HBOT, hyperbaric oxygen therapy; HD, hemodialysis; NA, not available; PD, peritoneal dialysis; RRT, renal replacement therapy; STS, sodium thiosulphate.

^a^ Age data available for 89 patients; sex available for 94 patients.

**Figure 2.**
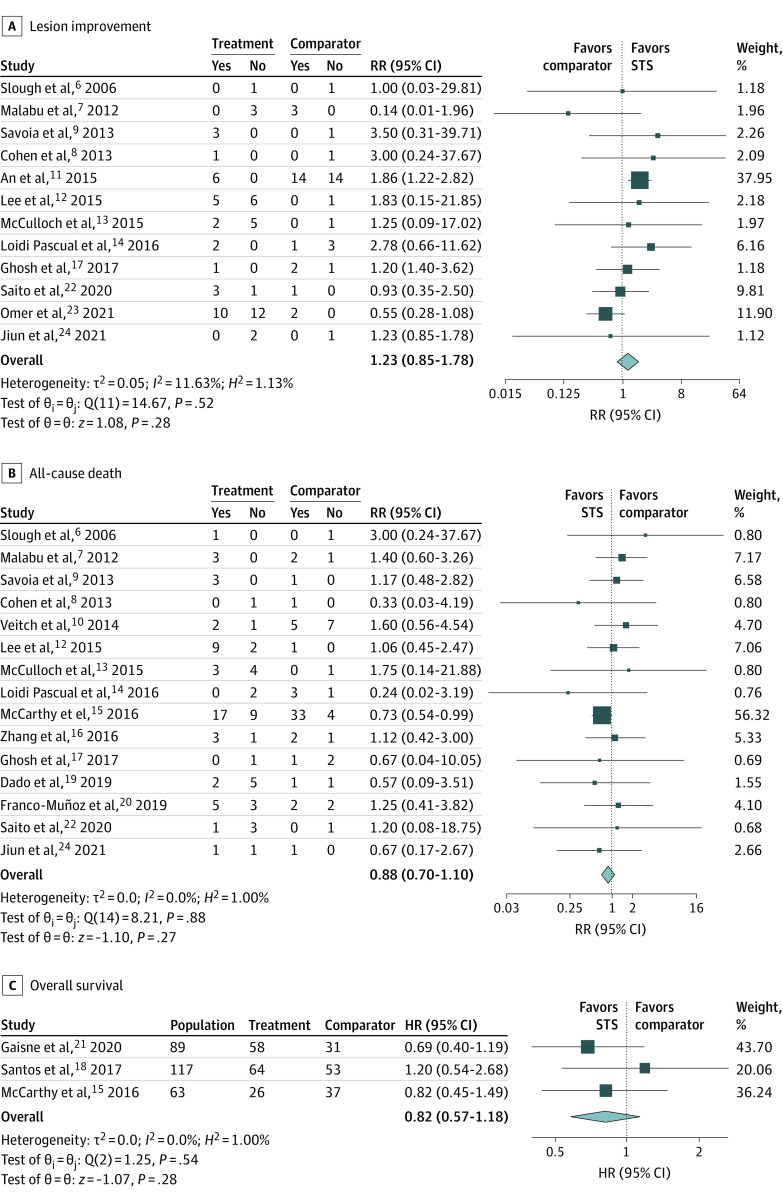
The Association of Sodium Thiosulphate (STS) With Lesion Improvement and Survival Among Patients With Calciphylaxis

No significant difference was observed for the outcome of skin lesion improvement (12 studies, 110 patients; RR, 1.23; 95% CI, 0.85-1.78) between the STS and the comparator groups, although the RR favored the STS group (Figure 2A). Similarly, despite a lower risk of death noted in the STS group in studies that provided dichotomous dead or alive outcome, no significant difference was achieved (15 studies, 158 patients; RR, 0.88; 95% CI, 0.70-1.10) (Figure 2B). None of the 3 studies that provided time-to-event death data showed an association between STS treatment and time to death. No significant benefit was observed after synthesis using meta-analysis (3 studies, 269 participants; hazard ratio, 0.82; 95% CI, 0.57-1.18) (Figure 2C). In meta-regression, the log RR of lesion improvement associated with STS was found to be negatively correlated with publication year (coefficient = −0.14; *P* = .008) (Table 2).

**Table 2.** Meta-regression for the Association of Publication Year With Outcomes of Calciphylaxis

Estimates^a^	Coefficient (95% CI)	Standard error	*z* Score	*P* value
Lesion improvement	−0.14 (−0.25 to −0.04)	0.05	−2.65	.008
RR for mortality	−0.09 (−0.20 to −0.03)	0.06	−1.50	.13
HR for mortality	−0.07 (−0.26 to −0.13)	0.10	−0.67	.50

Abbreviations: HR, hazard ratio; RR, risk ratio.

^a^ Correlation analysis between publication year and log effect size estimates using *z* test based on random-effects model (Empirical Bayes).

We performed sensitivity analyses on effect size measurements and single studies. Compared with RR, overall odds ratios (ORs) for lesion improvement and mortality were larger with wider 95% CIs, but no differences were noticed between the 2 groups (eFigure 1 in Supplement 1). In sensitivity analyses removing single studies, removing Omer et al^23^ resulted in a RR of 1.64 (95% CI, 1.17-2.29) in lesion improvement, which favored the use of STS, while removing An et al^11^ turned the RR to 0.99 (95% CI, 0.59-1.67) (eFigure 2A in Supplement 1). No change in the conclusion or direction for survival was noticed no matter which study was removed from the original analyses (eFigures 2B and 2C in Supplement 1). We synthesized small studies (total sample size fewer than 10) and large studies (total sample size of 10 or more) separately. No significant improvement in lesions or survival was observed in analyses limited to small or large studies (eFigure 3 in Supplement 1). Sensitivity analysis was also performed in studies only including dialysis patients. In these studies, RR for lesion improvement was 0.68 (95% CI, 0.41-1.14) while that for all-cause death was 1.15 (95% CI, 0.73-1.80) (eFigure 4 in Supplement 1). Both favored the comparator group, although there was no statistically significant association.

Based on the *I^2^* test, no obvious or high heterogeneity was noted in present analyses. Based on the ROBINS-I tool, the 19 cohort studies were evaluated to have a moderate to a critical risk of bias (eFigure 5 in Supplement 1). No small study effect was discovered (eFigure 6 in Supplement 1).

## Discussion

In this meta-analysis, to our knowledge the largest to date on this topic, we compared patients with CKD and calciphylaxis treated with STS and those without, and we observed no improvement in skin lesions or overall survival.

In case reports and multi-case reports, the effective rates for STS treatment were 67% and 84.4%, respectively.^25^ Complete and partial wound healing was observed in 80.3% of the patients receiving STS in case reports and case series, and 72.1% of those patients in cohort studies.^26^ However, the mortality rate remained high. In the largest cohort study of 172 patients treated with STS for calciphylaxis, the mortality rate was 35% at 1 year and 42% for the entire follow-up.^27^ Udomkarnjananun et al^26^ pooled data from 129 patients experiencing calciphylaxis in cohort studies with or without controls and found no difference in the risk of amputation, worsening of lesions, and death between patients treated with and without STS. Our study included more recently published studies and more patients than the former analysis. Sensitivity analysis and meta-regression were done to explore how various study characteristics may have factored into results. Notably, worse outcomes were observed in studies only focusing on dialysis patients. These findings raise questions regarding the role of STS in treating patients with calciphylaxis. However, treatment details about STS were not available from most of the studies and cannot be controlled by investigators. Notably, an overall survival improvement in patients with calciphylaxis has been reported with an effective therapeutic regimen of STS for not less than 2 weeks or with a cumulative dose of no less than 150 g.^21^ Meanwhile, negative correlation between skin lesion improvement and publication year implies a publication bias where successful treatment with STS was more likely to be published in the past, while more recently nonresponders have also been published. Thus, well-designed randomized controlled trials are warranted to establish STS’s effect on calciphylaxis.

However, recruitment of patients remains a challenge since calciphylaxis is a rare disease with life-threating nature. CALISTA trial (NCT03150420), a phase 3 trial designed to examine the effect of IV STS on acute calciphylaxis-associated pain was terminated early. An ongoing trial BEAT-CALCI (NCT05018221) is currently comparing STS, magnesium, and vitamin K treatments for patients with calciphylaxis.

### Limitations

This study had several limitations. Due to the lack of randomized clinical trials in this field, we gathered data only from cohort studies to do the analysis. Bias could arise from multiple sources, which was tested by the ROBINS-I tool. Of note, except for 2 studies (An et al^11^ and Omer et al^23^) that contributed the most weight to the analyses, the remaining studies were small and many had zero events of interest. Preexisting conditions could be unbalanced, as shown in Table 1. Important confounders, such as age, sex, and therapies like hyperbaric oxygen therapy were not balanced between the 2 groups. Moreover, information regarding potential additional therapies administered to patients was not consistently available, making reliable comparisons between STS and other therapies impossible. Our analysis might be underpowered and could yield residual confounding. Furthermore, lesion improvement may not be assessed uniformly throughout the studies. Another important outcome, pain intensity improvement, was not analyzed because it has not been consistently reported.

## Conclusion

Intravenous STS was not associated with skin lesion improvement or survival benefits in patients with CKD experiencing calciphylaxis in this meta-analysis. A future large and well-designed randomized clinical trial is warranted to establish the effect of STS on calciphylaxis. In addition, we also call for large prospective studies to investigate the patient characteristics that may be associated with improvement upon treatment with STS. Identification of such factors will allow judicious application of STS to patients with calciphylaxis and will guide the patient selection criteria of future clinical trials.

## Notes

Supplement 1. **eFigure 1.** Sensitivity Analysis for Effect Measurements **eFigure 2.** Sensitivity Analysis by Removing Single Studies **eFigure 3.** Sensitivity Analysis of Small or Large Studies **eFigure 4.** Sensitivity Analysis for Studies Only Including Dialysis Patients **eFigure 5.** Risk-of-Bias Assessment of the 19 Cohort Studies Using ROBINS-I Tool **eFigure 6.** Funnel Plots and Egger Test *P* Values for Meta-analyses in the Study **eTable 1.** The Controlled Vocabulary Terms and Synonyms **eTable 2.** Complete Search Strategy

Supplement 2.
Data Sharing Statement
