# Supplementary material for: Intravenous Sodium Thiosulphate for Calciphylaxis of Chronic Kidney Disease: A Systematic Review and Meta-analysis
Source: JAMA Netw Open. 2023 Apr 26;6(4):e2310068. doi: 10.1001/jamanetworkopen.2023.10068 (PMC10134003; doi:10.1001/jamanetworkopen.2023.10068)
Supplement: Supplement 1. — eFigure 1. Sensitivity Analysis for Effect Measurements eFigure 2. Sensitivity Analysis by Removing Single Studies eFigure 3. Sensitivity Analysis of Small or Large Studies eFigure 4. Sensitivity Analysis for Studies Only Including Dialysis Patients eFigure 5. Risk-of-Bias Assessment of the 19 Cohort Studies Using ROBINS-I Tool eFigure 6. Funnel Plots and Egger Test P Values for Meta-analyses in the Study eTable 1. The Controlled Vocabulary Terms and Synonyms eTable 2. Complete Search Strategy [file jamanetwopen-e2310068-s001.pdf]

Supplemental Online Content

Wen W, Portales-Castillo I, Seethapathy R, et al. Intravenous sodium thiosulphate for calciphylaxis of chronic kidney disease. *JAMA Netw Open.* 2023;6(4):e2310068. doi:10.1001/jamanetworkopen.2023.10068

eFigure 1. Sensitivity Analysis for Effect Measurements

eFigure 2. Sensitivity Analysis by Removing Single Studies

eFigure 3. Sensitivity Analysis of Small or Large Studies

eFigure 4. Sensitivity Analysis for Studies Only Including Dialysis Patients

eFigure 5. Risk-of-Bias Assessment of the 19 Cohort Studies Using ROBINS-I Tool

eFigure 6. Funnel Plots and Egger Test *P* Values for Meta-analyses in the Study

eTable 1. The Controlled Vocabulary Terms and Synonyms

eTable 2. Complete Search Strategy

This supplemental material has been provided by the authors to give readers additional information about their work.

© 2023 Wen W et al. *JAMA Network Open.*

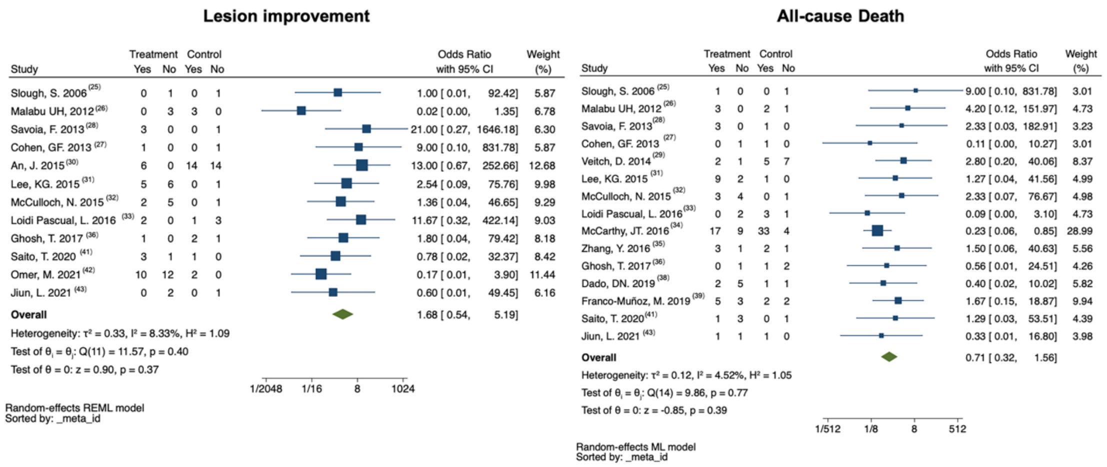

eFigure 1: Sensitivity analysis for effect measurements.

© 2023 Wen W et al. JAMA Network Open.

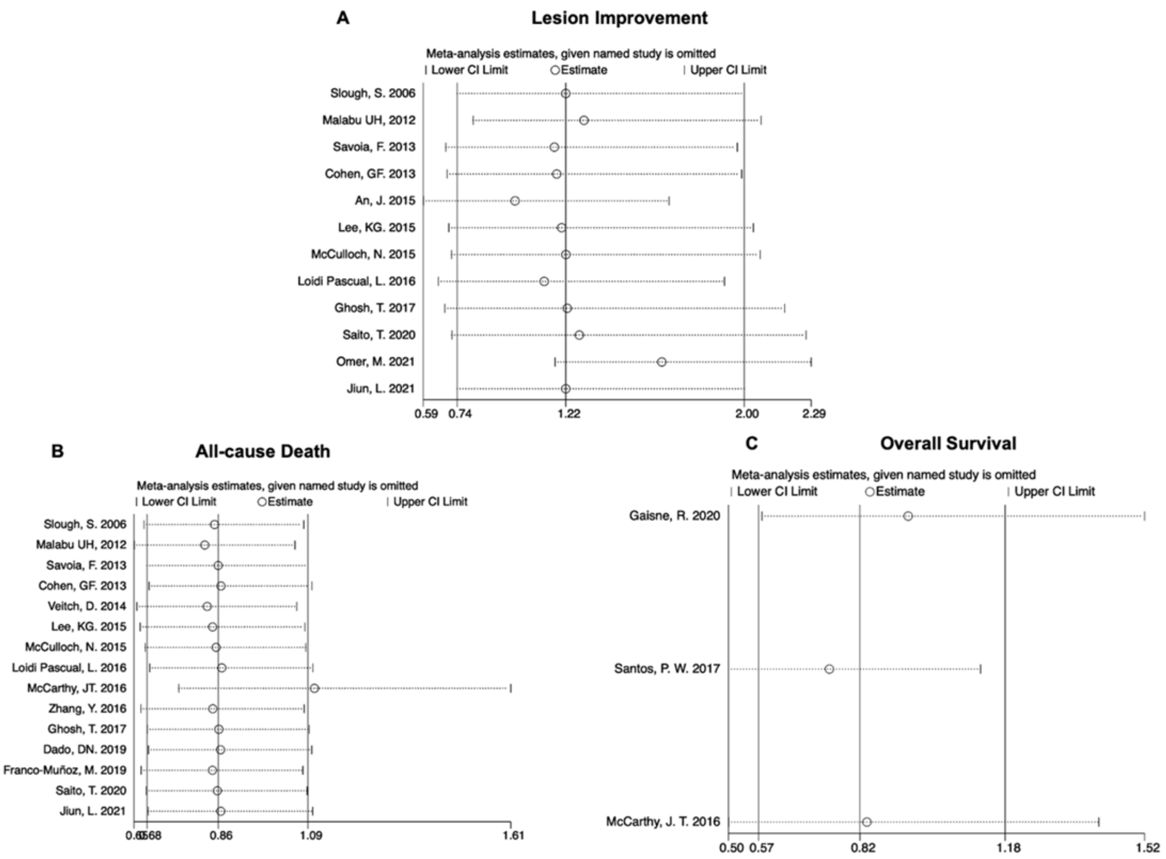

eFigure 2: Sensitivity analysis by removing single studies. Removing Omer, M., et al. 2021 resulted in a RR of 1.64 (95%CIs: 1.17, 2.29) in lesion improvement which favored the use of STS (A).

© 2023 Wen W et al. *JAMA Network Open.*

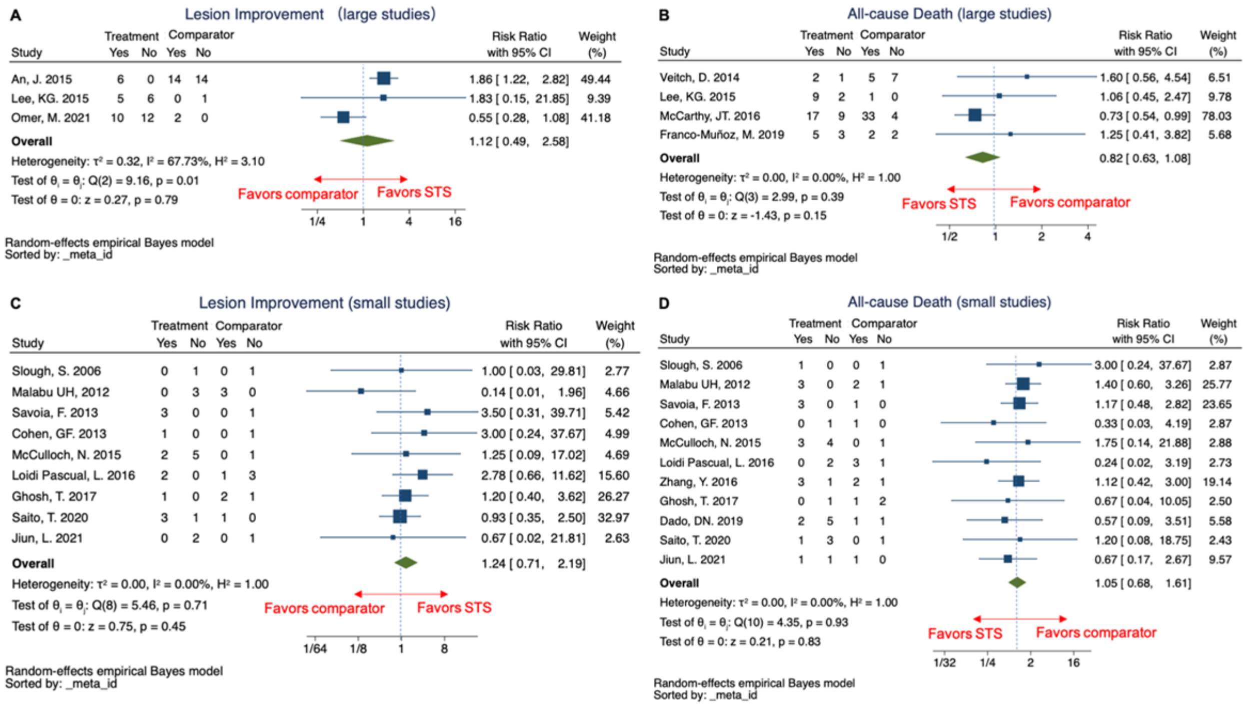

eFigure 3: Sensitivity analysis of small or large studies. No significant difference was found for lesion 64 improvement and all-cause death between calciphylaxis patients treated with and without STS either in small studies (sample size<10) or in large studies (sample size≥10).

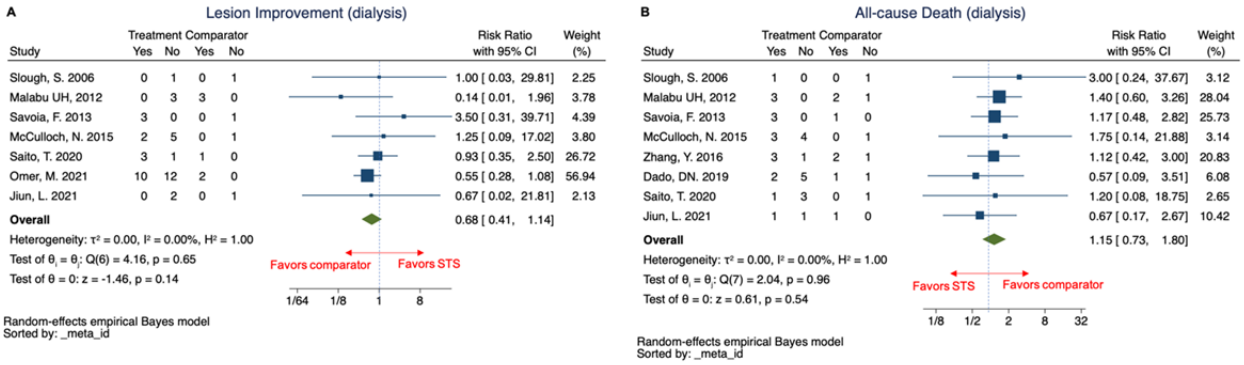

eFigure 4: Sensitivity analysis for studies only included dialysis patients. No significant difference was found for lesion improvement and all-cause death between calciphylaxis patients treated with and without STS in studies only included dialysis patients.

© 2023 Wen W et al. *JAMA Network Open.*

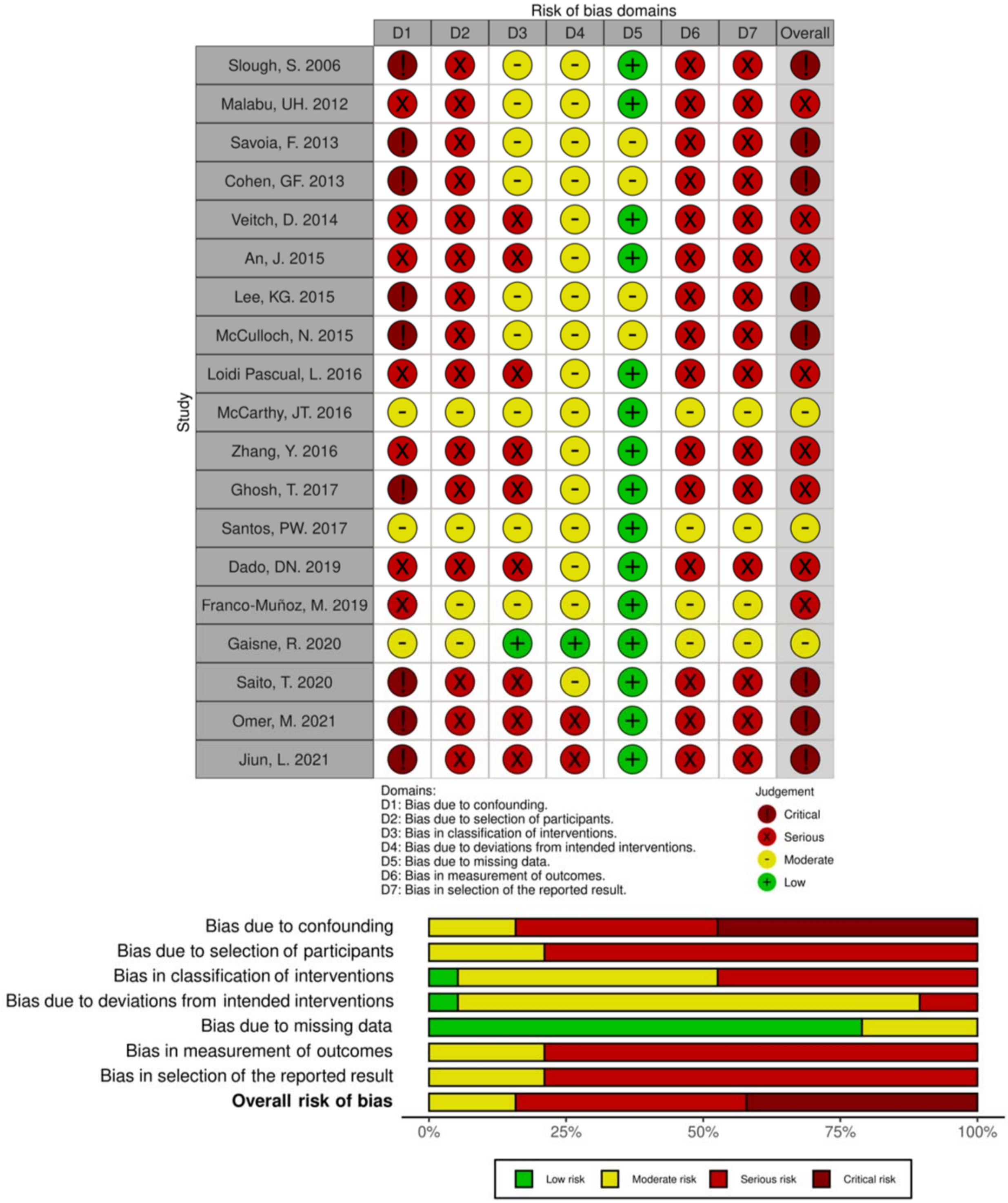

eFigure 5: Risk-of-bias assessment of the 19 cohort studies using ROBINS-I tool.

© 2023 Wen W et al. *JAMA Network Open.*

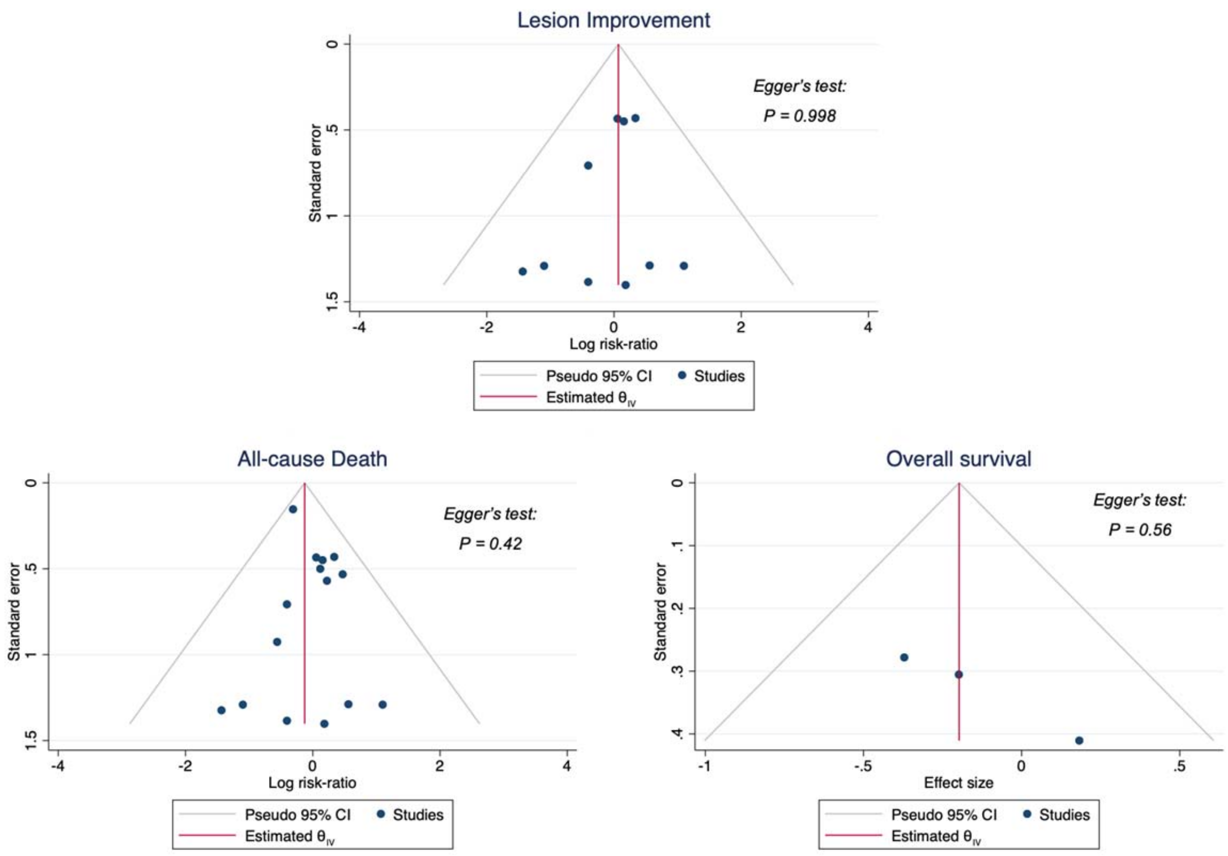

eFigure 6: Funnel plots and Egger's test P values for meta-analyses in the study.

© 2023 Wen W et al. *JAMA Network Open.*

eTable 1: The controlled vocabulary terms and synonyms

	PATIENT/POPULATION	INTERVENTION
**Controlled Vocabulary Term (MeSH/ Emtree)**	Vascular Calcification<br>Cardiovascular calcification<br>Calciphylaxis<br>Calcification of Joints and Arteries [Supplementary Concept]<br>Aortic Valve, Calcification of [Supplementary Concept]	Sodium thiosulfate<br>Thiosulfates
**Synonyms**	Calciphylax*;<br>Calcific uremic arteriolopathy;<br>Calci*;<br>Microcalci*	thiosulfuric acid; disodium salt; hyposulfite; nsc 45624; s hydril; sodium hyposulfite; sodium thiosulphate; sodothiol; sulfactol; sulfothiorine; thiosulfate sodium; thiosulphate sodium

© 2023 Wen W et al. *JAMA Network Open.*

eTable 2: Complete search strategy

**Pubmed**	("Thiosulfates"[MeSH Terms] OR "thiosulfates/pharmacology"[MeSH Terms] OR "sodium thiosulfate"[Supplementary Concept] OR "disodium salt"[Tiab] OR "sodium thiosulfate"[Tiab] OR "hyposulfite"[Tiab] OR "nsc 45624"[Tiab] OR "s hydril"[Tiab] OR "sodium hyposulfite"[Tiab] OR "sodium thiosulphate"[Tiab] OR "sodothiol"[Tiab] OR "sulfactol"[Tiab] OR "sulfothiorine"[Tiab] OR "thiosulfate sodium"[Tiab] OR "thiosulphate sodium"[Tiab]) AND ("Vascular Calcification"[MeSH Terms] OR "Calciphylaxis"[MeSH Terms] OR "Calcification of Joints and Arteries"[Supplementary Concept] OR "aortic valve calcification of"[Supplementary Concept] OR Calciphylax*[Tiab] OR Idiopathic Calciphylax*[Tiab] OR Calcific uremic arteriolopathy[Tiab] OR Calci*[Tiab] OR Microcalci*[Tiab])
**Embase**	('sodium thiosulfate'/exp OR 'sodium thiosulfate' OR 'sodium thiosulfate*' OR 'thiosulfate*' OR 'thiosulfuric acid' OR 'disodium salt' OR 'hyposulfite'/exp OR 'hyposulfite' OR 'nsc 45624'/exp OR 'nsc 45624' OR 's hydril'/exp OR 's hydril' OR 'sodium hyposulfite'/exp OR 'sodium hyposulfite' OR 'sodium thiosulphate'/exp OR 'sodium thiosulphate' OR 'sodothiol'/exp OR 'sodothiol' OR 'sulfactol'/exp OR 'sulfactol' OR 'sulfothiorine'/exp OR 'sulfothiorine' OR 'thiosulfate sodium'/exp OR 'thiosulfate sodium' OR 'thiosulphate sodium'/exp OR 'thiosulphate sodium') AND [embase]/lim AND ('cardiovascular calcification'/exp OR 'calciphylaxis'/exp OR 'calciphylax*':ti,ab OR 'calci*':ti,ab OR 'microcalci*':ti,ab) AND [embase]/lim
**Cochrane Library**	([Thiosulfates][MeSH Terms] OR (Thiosulfates) OR (disodium salt) OR (sodium thiosulfate) OR (hyposulfite) OR (nsc 45624) OR (s hydril) OR (sodium hyposulfite) OR (sodium thiosulphate) OR (sodothiol) OR (sulfactol) OR (sulfothiorine) OR (thiosulfate sodium) OR (thiosulphate sodium)) AND ([Vascular Calcification] [MeSH Terms] OR [Calciphylaxis][MeSH Terms] OR (Calciphylax*) OR (Idiopathic Calciphylax*) OR (Calcific uremic arteriolopathy) OR (Calci*)OR (Microcalci*)
**Web of Science**	(TS=((Thiosulfates) OR (sodium thiosulfate) OR (disodium salt) OR (sodium thiosulfate) OR (hyposulfite) OR (nsc 45624) OR (s hydril) OR (sodium hyposulfite) OR (sodium thiosulphate) OR (sodothiol) OR (sulfactol) OR (sulfothiorine) OR (thiosulfate sodium) OR (thiosulphate sodium))) AND (TS=((Calciphylax*) OR (Calcific uremic arteriolopathy) OR (Calci*) OR (Microcalci*)))

© 2023 Wen W et al. *JAMA Network Open.*
