# Supplementary material for: Intravenous Sodium Thiosulphate for Calciphylaxis of Chronic Kidney Disease: A Systematic Review and Meta-analysis
Source: JAMA Netw Open. 2023 Apr 26;6(4):e2310068. doi: 10.1001/jamanetworkopen.2023.10068 (PMC10134003; doi:10.1001/jamanetworkopen.2023.10068)
Supplement: Supplement 2. — Data Sharing Statement [file jamanetwopen-e2310068-s002.pdf]

Data Sharing Statement

Wen. Intravenous Sodium Thiosulphate for Calciphylaxis of Chronic Kidney Disease. *JAMA Netw Open*. Published April 26, 2023. doi:10.1001/jamanetworkopen.2023.10068

Data
Data available: Yes
Data types: Other (please specify)
Additional Information: Data extraction forms
How to access data: wwa01275@btch.edu.cn
When available: With publication

Supporting Documents
Document types: None

Additional Information
Who can access the data: researchers whose proposed use of the data has been approved
Types of analyses: For future study
Mechanisms of data availability: after approval of a proposal,
Any additional restrictions: Only for research use
